# Quantitative traits loci mapping and molecular marker development for total glutenin and glutenin fraction contents in wheat

**DOI:** 10.1186/s12870-021-03221-0

**Published:** 2021-10-06

**Authors:** Zhengfu Zhou, Ziwei Zhang, Annaliese S. Mason, Lingzhi Chen, Congcong Liu, Maomao Qin, Wenxu Li, Baoming Tian, Zhengqing Wu, Zhensheng Lei, Jinna Hou

**Affiliations:** 1 Henan Institute of Crop Molecular Breeding, Henan Academy of Agricultural Sciences, Zhengzhou, 450002 China; 2 Agronomy College, Zhengzhou University, 450001 Zhengzhou, China; 3 National Key Laboratory of Wheat and Maize Crop Science, Henan Agricultural University, Zhengzhou, 450002 China; 4 Chair of Plant Breeding, Institute of Crop Science and Resource Conservation, University of Bonn, Bonn, Germany

**Keywords:** Glutenin content, High- and low-molecular-weight glutenin subunits, End-use quality, QTL mapping, KASP marker, Wheat

## Abstract

**Background:**

Glutenin contents and compositions are crucial factors influencing the end-use quality of wheat. Although the composition of glutenin fractions is well known, there has been relatively little research on the genetic basis of glutenin fractions in wheat.

**Results:**

To elucidate the genetic basis for the contents of glutenin and its fractions, a population comprising 196 recombinant inbred lines (RILs) was constructed from two parents, Luozhen No.1 and Zhengyumai 9987, which differ regarding their total glutenin and its fraction contents (except for the By fraction). Forty-one additive Quantitative Trait Loci (QTL) were detected in four environments over two years. These QTL explained 1.3% - 53.4% of the phenotypic variation in the examined traits. Forty-three pairs of epistatic QTL (E-QTL) were detected in the RIL population across four environments. The QTL controlling the content of total glutenin and its seven fractions were detected in clusters. Seven clusters enriched with QTL for more than three traits were identified, including a QTL cluster *6AS-3*, which was revealed as a novel genetic locus for glutenin and related traits. Kompetitive Allele-Specific PCR (KASP) markers developed from the main QTL cluster *1DL-2* and the previously developed KASP marker for the QTL cluster *6AS-3* were validated as significantly associated with the target traits in the RIL population and in natural varieties.

**Conclusions:**

This study identified novel genetic loci related to glutenin and its seven fractions. Additionally, the developed KASP markers may be useful for the marker-assisted selection of varieties with high glutenin fraction content and for identifying individuals in the early developmental stages without the need for phenotyping mature plants. On the basis of the results of this study and the KASP markers described herein, breeders will be able to efficiently select wheat lines with favorable glutenin properties and develop elite lines with high glutenin subunit contents.

**Supplementary Information:**

The online version contains supplementary material available at 10.1186/s12870-021-03221-0.

## Background

Glutenins consist of high- and low-molecular-weight subunits (HMW-GS and LMW-GS), which are linked by disulfide bonds to form polymeric proteins in wheat [1]. The HMW-GS content is highly associated with dough viscoelasticity and bread-baking quality, despite only accounting for only about 7%-15% of the total protein in flour [2]. Additionally, HMW-GSs are encoded by three homologous loci located on the long arm of chromosome 1 in each genome (*Glu-A1*, *Glu-B1* and *Glu-D1*) which contribute two tightly linked genes for x- and y- type subunits that are distinguished by molecular weights and the conserved N~terminal domain sequences [3, 4]. The LMW-GSs, which account for 60% of the total glutenins, are also major determinants of viscoelastic properties of dough [5]. The genetic loci related to LMW-GSs are *Glu-A3*, *Glu-B3* and *Glu-D3*, which are located on the short arm of chromosome 1. In addition to these homologous loci, three new loci, *Glu-2*, *Glu-4* and *Glu-5*, have been identified on chromosomes 1B, 1D and 7D, respectively [6, 7].

The various alleles at the HMW-GS and LMW-GS loci are responsible for the diverse combinations of subunits that contribute to the end-use quality of wheat [8]. For example, 1Dx5 + 1Dy10 (*Glu-D1d*), 1Bx7 + 1By8 (*Glu-B1b*), and 1Bx17 + 1By18 (*Glu-B1i*) lead to higher quality, whereas 1Dx2 + 1Dy12 (*Glu-D1a*), 1Bx20 (*Glu-B1e*), and 1Bx7 + 1By9 (*Glu-B1c*) are related to poor quality [9–12]. Interestingly, there are interactions between the *Glu-1* loci and *Glu-3* loci, implying there is a complex network controlling the composition and content of glutenin in wheat grains [13]. Additionally, modifying the contents of certain glutenin fractions induces properties changes in dough. More specifically, the absence of *Dx2* decreases the dough quality through delaying glutenin polymerization [14]. In contrast, the over-expression of *Ax1*, *Dx5* and *Dy10* significantly increases glutenin polymers so as to improve the strength and elasticity of dough. Applying transgenic technologies to silence *Dx5* significantly decreases the Zeleny sedimentation value, gluten index, and dough formation time and stability [15].

Quantitative trait loci (QTL) associated with the total glutenin, HMW-GS and LMW-GS contents have been detected, and the *Glu-A1*, *Glu-B1*, *Glu-A3* and *Glu-B3* genetic loci have been elucidated [16]. The QTL for *Glu-B1x*, *Glu-D1x* and *Glu-D1y* were mapped on chromosome 5A in wheat [17]. However, there have been relatively few studies on the QTL for these traits in wheat, with investigations restricted to low density markers in only a few environments. Little research has also been done to systematically identify genetic loci corresponding to the x- and y- type subunits of HMW-GS and to evaluate the allelic contributions in natural varieties. In the present study, a recombinant inbred line (RIL) population constructed from two parents, Luozhen No. 1 and Zhengyumai 9987, which differ regarding the quality and content of glutenin fractions, was used for dissecting QTL associated with total glutenin and seven fractions (HMW-GS, LMW-GS, Ax, Bx, By, Dx, and Dy) in four environments. We excluded Ay because the corresponding gene is usually silenced [18]. These QTL preferentially clustered in specific chromosomal regions. We subsequently developed and tested molecular markers for the two main QTL clusters. This research highlights the content of different glutenin fractions as a new aspect and strategy for quality improvement in wheat.

## Results

### Phenotypic variation and genetic effects of the parents and RILs

**Fig. 1:**
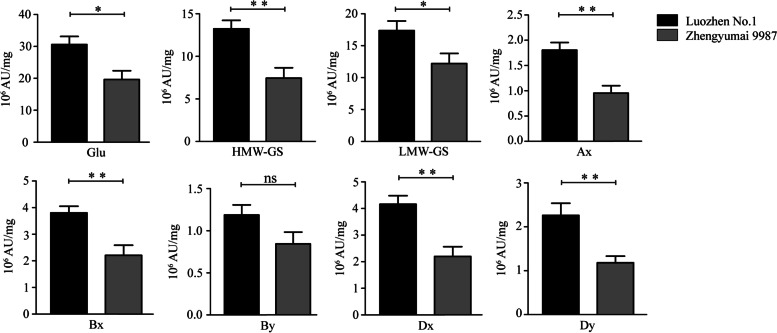
Phenotypic divergence between the two parents in four environments. The average values of each fraction are indicated on the y-axis. Significant differences between the parents are indicated (* P < 0.05 and ** P < 0.01). Only the By content did not differ significantly between the two parents of the RIL population

**Fig. 2:**
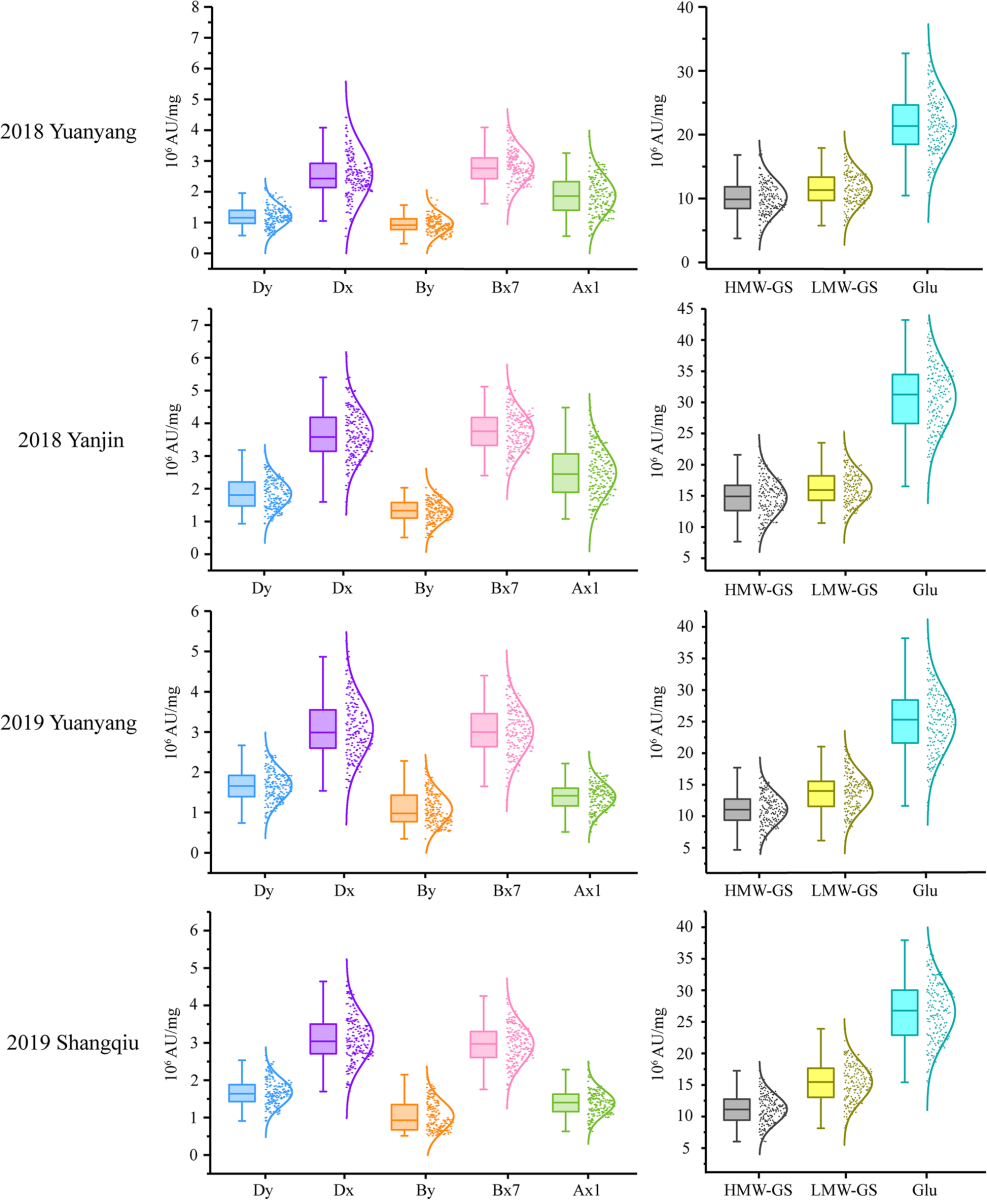
Phenotypic variations of each glutenin fraction in the RIL population in different environments. The phenotypic distribution of each glutenin fraction in the RIL population in four environments is presented. The four different environments are indicated on the left side and the different colored dots and bars represent different traits

The contents of total glutenin and its seven fractions Ax, Bx, By, Dx, Dy, HMW-GS and LMW-GS were investigated in the parents and the RIL population under four environments (Table S1). The parent Luozhen No.1 had approximately twice as much glutenin as Zhengyumai 9987 (Fig. 1 and Table S2). The total glutenin and glutenin fractions (except for By) contents differed significantly between the two parents. The Ax, Bx, Dx, Dy and HMW-GS contents differed at the P < 0.01 level, whereas LMW-GS and total glutenin contents differed at the P < 0.05 level (Fig. 1 and Table 1). All traits were normally distributed in the RIL population (Fig. 2). In the two 2018 environments, the rank order for the relative abundance of each fraction was Bx> Dx > Ax> Dy > By, whereas in 2019, the rank order for the relative abundance was Bx > Dx > Dy > Ax > By in Yuanyang and Dx > Bx > Dy > Ax > By in Shangqiu (Fig. 2 and Table S2). The effects of the genotype, the environment and genotype-by-environment interactions significantly influenced all traits (P < 0.001) (Table 1). The broad-sense heritability of the traits was higher than 65%, with the exception of the By content, which was only 39.1% heritable (Table 1). All traits were significantly positively correlated with each other in all environments (P < 0.01). The correlation coefficient (*r*) between traits varied from 0.268 to 0.909. The highest correlation (*r* = 0.909) was between HMW-GS and Ax. The correlation between total glutenin and the glutenin fractions were lower (*r* =0.484 - 0.891) (Table S3).

**Table 1:** Phenotypic variance components for each glutenin fraction across multiple environments

Traits	ANOVA^a^				
	$\sigma_{\textbf{G}}^{\textbf{2}}$	$\sigma_{\textbf{E}}^{\textbf{2}}$	$\sigma_{\textbf{GE}}^{\textbf{2}}$	$\sigma_{\textbf{e}}^{\textbf{2}}$	*H*^2^
Ax	0.76^***^	52.76^***^	0.15^***^	0.124	67.54
Bx	0.89^***^	33.60^***^	0.17^***^	0.146	81.39
By	0.29^***^	5.21^***^	0.10^***^	0.026	39.12
Dx	1.50^***^	40.27^***^	0.31^***^	0.054	76.90
Dy	0.45^***^	15.34^***^	0.07^***^	0.012	87.43
HMW-GS	20.62^***^	791.01^***^	2.71^***^	0.763	88.00
LMW-GS	22.54^***^	848.97^***^	3.55^***^	0.549	84.14
Glu	69.56^***^	2839.37^***^	9.75^***^	1.401	87.56

^a^ Variance contributed by genotype ($\mathrm{\sigma_G^2}$), the environment ($\mathrm{\sigma_E^2}$), genotype-by-environment interactions ($\mathrm{\sigma_{GE}^2}$), errors ($\mathrm{\sigma_e^2}$) and broad-sense heritability (*H*^2^). ^***^ Variances contributed by genotype, the environment, and genotype-by-environment interactions were significant (P < 0.001)

### Genetic loci controlling the total glutenin and its fraction contents

**Fig. 3:**
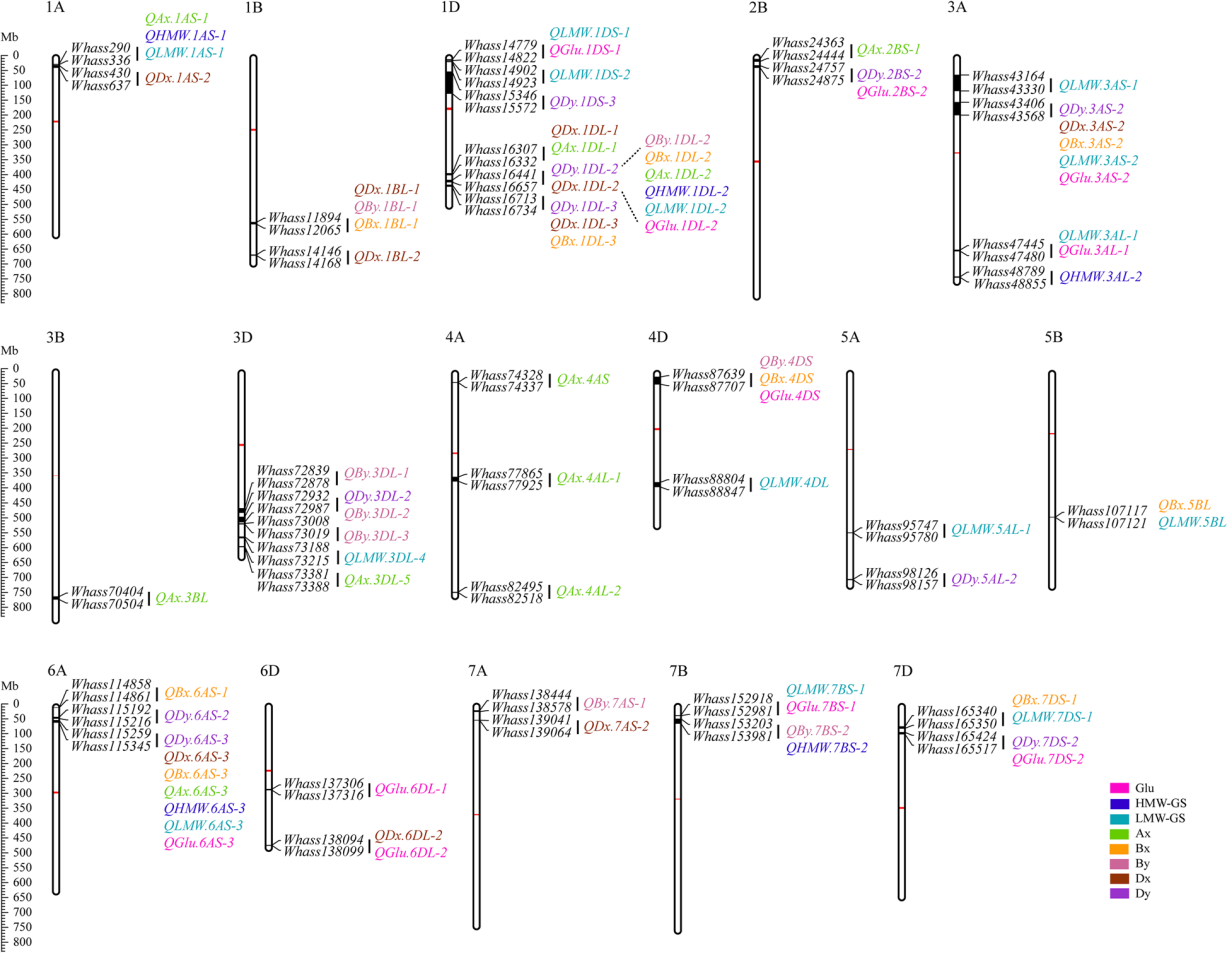
Distribution of QTL associated with total glutenin and its fractions content on the physical map. The QTL represented by black bars are marked according to the physical position on the map. The physical positions are indicated to the left of the first chromosome in each row. The marker names are provided to the right of each chromosome. The QTL associated with different traits are assigned different colors (legend in the lower right corner). The red bars indicate the centromeres positions

A total of 41 additive QTL related to the total glutenin and its fraction contents were detected (Fig. 3 and Table S4). These QTL were distributed on 16 chromosomes: 1A (2 QTL), 1B (2 QTL), 1D (6 QTL), 2B (2 QTL), 3A (4 QTL), 3B (1QTL), 3D (5 QTL), 4A (3 QTL), 4D (2 QTL), 5A (2 QTL), 5B (1 QTL), 6A (3 QTL), 6D (2 QTL), 7A (2 QTL), 7B (2 QTL) and 7D (2 QTL) (Fig. 3, Table S4 and Figure S1-7). Individual QTL explained 1.3%-53.4% of the phenotypic variation, with the logarithm of the odds (LOD) ranging from 2.54 to 39.7 (Table S4). Regarding the individual traits, 10 (Ax), 7 (Bx), 8 (By), 10 (Dx), 9 (Dy), 4 (HMW-GS), 14 (LMW-GS) and 11 (total glutenin) QTL were detected (Table S4 and Figure S4).

The eleven QTL related to the total glutenin content were detected on chromosomes 1D (2 QTL), 2B (1 QTL), 3A (2 QTL), 4D (1 QTL), 6A (1 QTL), 6D (2 QTL), 7B (1 QTL) and 7D (1 QTL), respectively, explained 2.3%-26.9% of the phenotypic variation. Four QTL, *QGlu.1DL-2*, *QGlu.3AS-2*, *QGlu.4DS* and *QGlu.6AS-3*, were repeatedly detected in more than three environments (BLUP included) (Table S4 and Figure S1). Some environment-specific QTL were also detected, among which six and one were detected only in E2 and E3, respectively (Table S4 and Figure S1).

Four HMW-GS related QTL were detected, among which two (*QHMW.1DL-2* and *QHMW.6AS-3*) were identified in all environments, one (*QHMW.1AS-1*) was identified in two environments (BLUP included), and another (*QHMW.3AL-2*) was E2-specific (Table S4 and Figure S2). Individual loci contributed 4.0%-24.4 % to the phenotypic variation. However, the five fractions, Ax, Bx, By, Dx, and Dy, seemed quite susceptible to environmental conditions. Although there were 10, 7, 8, 9 and 9 QTL associated with Ax, Bx, By, Dx and Dy, respectively, most of these were environment-specific QTL. Hence, only nine QTL were repeatedly detected in more than three environments (BLUP included) (Table S4 and Figures S4-7). One of the QTL located in the *Whass16441*-*Whass16657* marker interval on chromosome 1D was associated with all glutenin fractions and contributed up to 53.4% of the phenotypic variation in the By content (Fig. 3 and Table S4).

Fourteen QTL associated with LMW-GS were distributed on 10 chromosomes (Figure S3). Four of these QTL (*QLMW.1DS-1*, *QLMW.1DL-2*, *QLMW.3AS-2* and *QHMW.6AS-3*) explained 3.1% - 22.2% of the phenotypic variation with a LOD of 2.66-15.28 and were detected in more than three environments (BLUP included). The other QTL were specific to certain environments (Table S4 and Figure S3).

### Detection of epistatic QTL for all traits

**Fig. 4:**
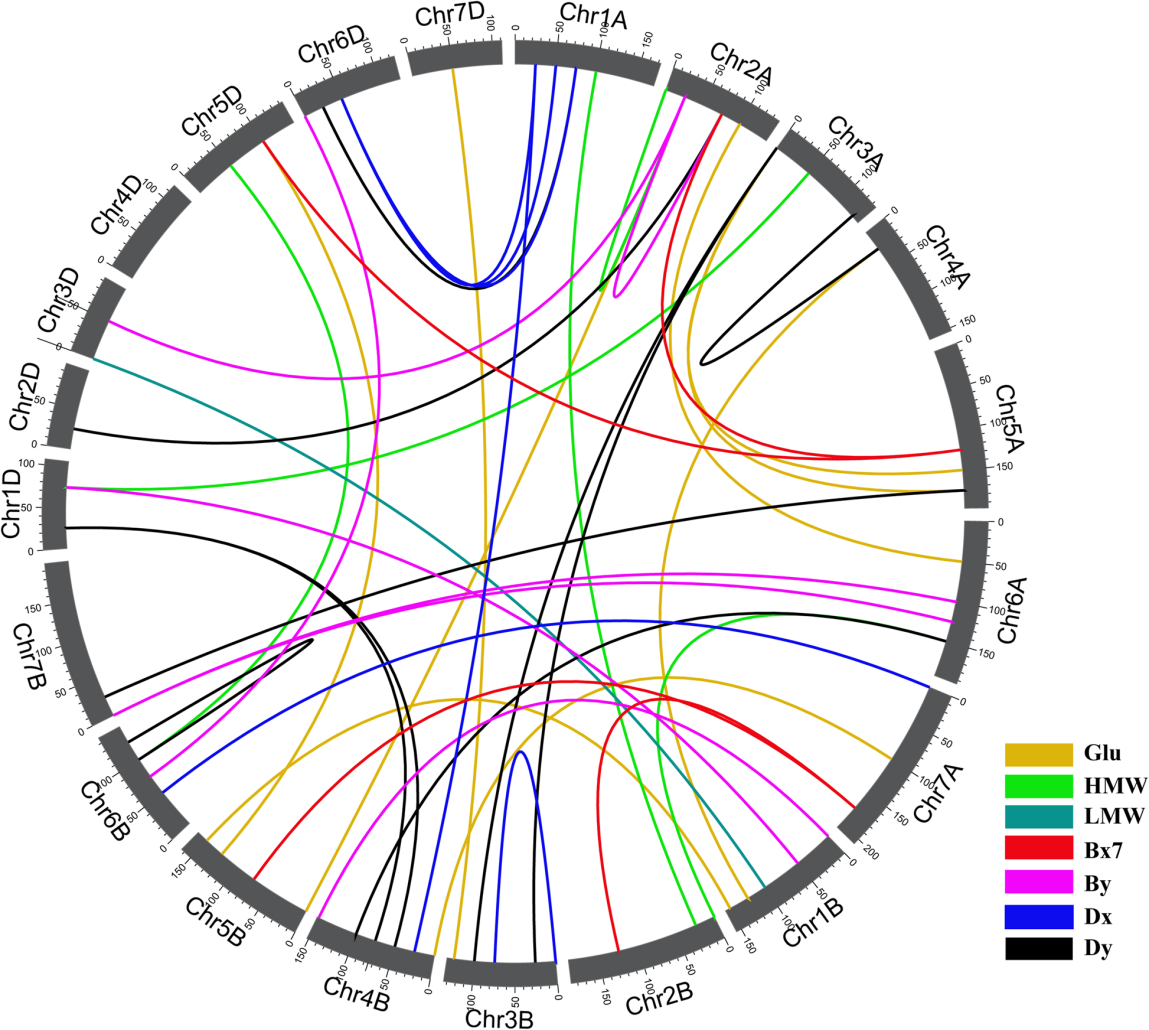
E-QTL for total glutenin and its fractions in the RIL population across four environments. The E-QTL interval pairs are linked by curves. Seven colors were used to represent the E-QTL for different glutenin fractions (legends in the lower right corner). Chromosomes are represented by grey bars. The bar length reflects the relative length of each chromosome

Forty-three pairs of epistatic QTL (E-QTL) were detected for total glutenin and its fractions in the RIL population across four environments (Table S5 and Fig. 4). More specifically, 9, 5, 1, 10, 7, 7 and 4 E-QTL detected for total glutenin, HMW-GS, LMW-GS, Dy, By, Dx and Bx, respectively. These E-QTL were estimated to explain 2.61% – 11.18% of the phenotypic variation. No E-QTL was detected for Ax (Table S5 and Fig. 4). Of the E-QTL, seven were detected in two environments, whereas two were revealed in three environments (Table S5). The QTL interval flanked by the molecular markers *Whass24363* and *Whass24444* on chromosome 2B revealed for total glutenin and Ax (*QGlu.2BS-2*and *QAx1.2BS-1* in E2) was estimated involving the interaction with the QTL region anchored between *Whass120631* and *Whass120491* on chromosome 6A.

### Dissection of the QTL clusters

**Fig. 5:**
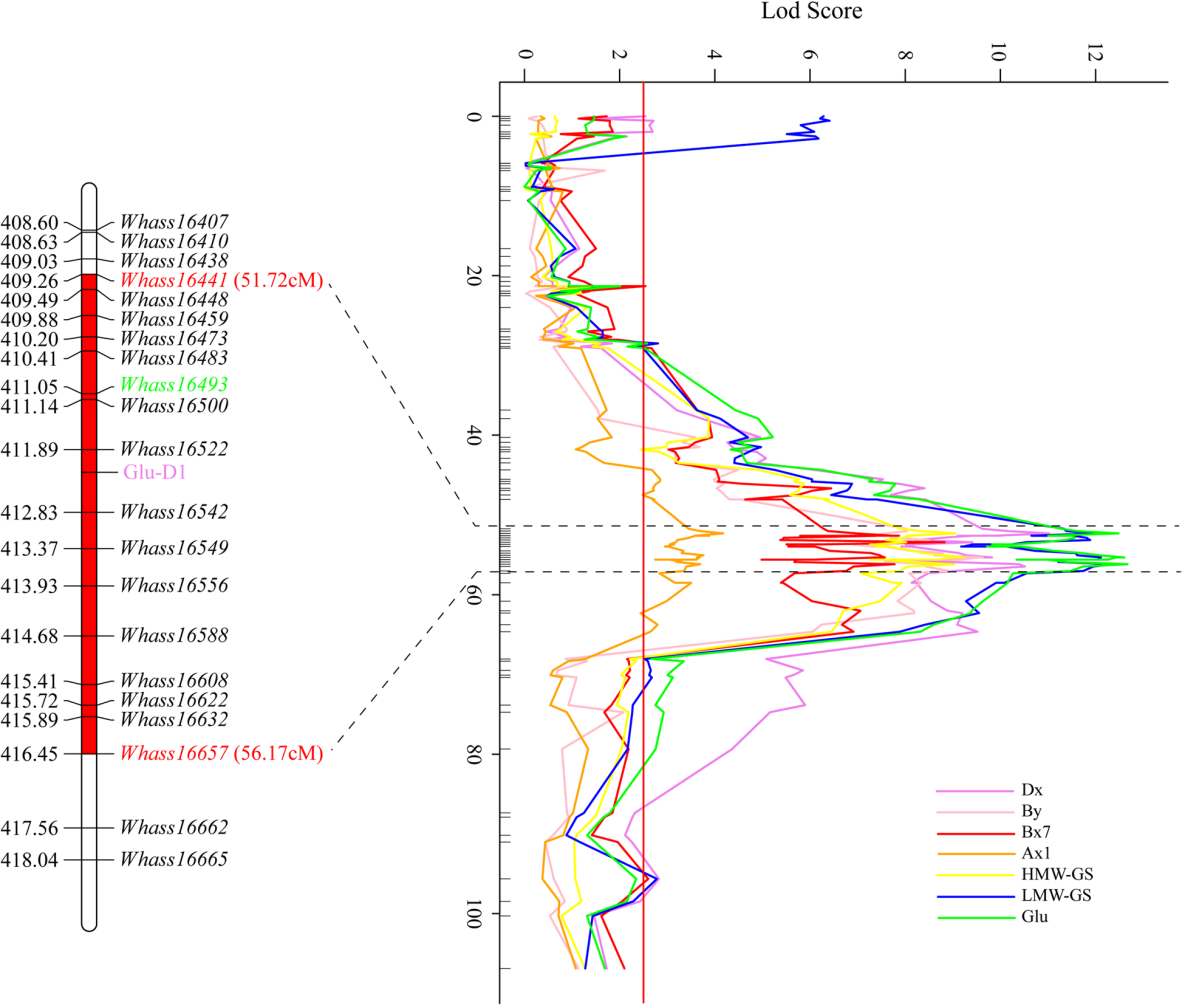
QTL cluster for total glutenin and its fraction contents detected on 1DL. The QTL cluster on 1DL was associated with all eight investigated traits. Curves with different colors indicate different traits. Molecular markers around the peak of the cluster and their corresponding genetic positions are labeled. The major locus for controlling the glutenin content, *Glu-D1* (purple), was mapped in this cluster. Two SNPs flanking the cluster, which were used for developing and validating KASP markers are in red

All forty-one QTL were mapped on the physical map constructed according to the sequence information derived from the specific locus amplified fragment (SLAF) tag and aligned with the reference genome sequence. Some QTL controlling the total glutenin and its fraction contents tended to co-localize on particular chromosomes (Fig. 3 and Figures S8-11). Seven regions enriched with QTL for more than three traits were designated as QTL clusters, which were distributed on the regions of 1AS-1, 1BL-1, 1DL-2, 1DL-3, 3AS-2, 4DS and 6AS-3 (Fig. 3 and Figures S8-11). The *1DL-2* QTL cluster controlled all eight traits (Fig. 5), whereas *6AS-3* included QTL affecting seven traits; the exception was the By fraction content (Fig. 3).

### Candidate gene analysis for the two main QTL clusters

A total of 164 annotated genes were identified from the two main QTL clusters, *1DL-2* and *6AS-3*, with the chromosome region on 1DL (409.26-416.45Mb) and 6AS (44.02-50.35Mb) (Table S6). Among them, 112, 53, 49 and 103 annotated genes had identified in the GO, KEGG, KOG and Swiss-prot database, respectively (Table S6). By GO analysis, 22 genes were detected in the cellular component category, 37 genes in the molecular function category, and 19 genes in the biological process category (Table S6 and Figure S12). Through KEGG analysis, 53 genes were detected in 29 pathways (Table S6 and Figure S13). In the KOG analysis, only two genes had unknown function (Table S6 and Figure S14). Among other genes, eight genes were related with posttranslational modification, protein turnover and chaperones, and four of them had a relation to transcription.

### Contribution of the two main QTL clusters to phenotypic variation

**Fig. 6:**
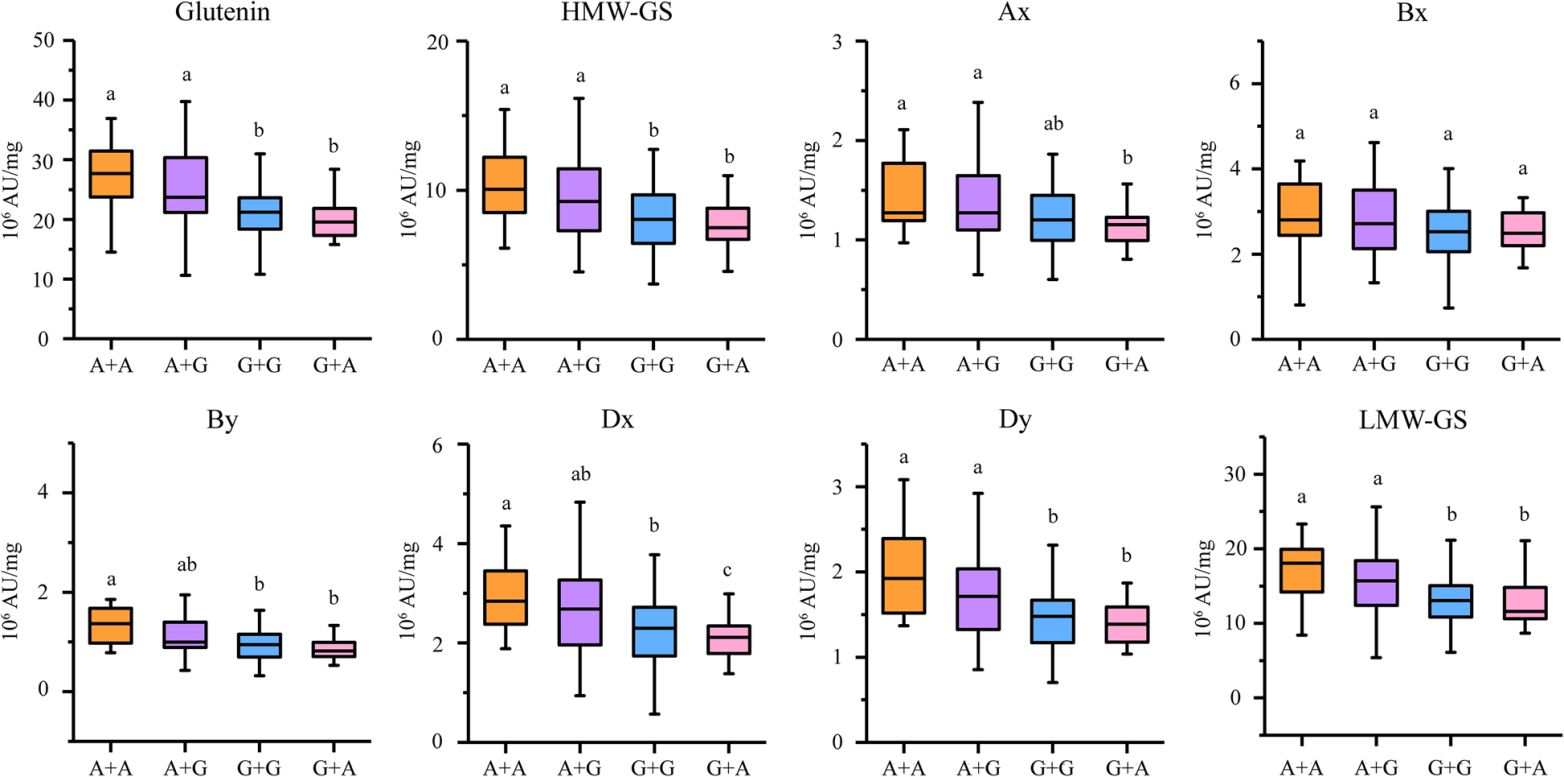
Effects of two SNP loci on glutenin fractions in a set of bread wheat cultivars. The effects of two SNPs on the content of glutenin fractions in 207 wheat varieties are presented. The genotypes labeled under each chart are for the SNPs *Whaas16493* and *Whaas115399*. The y-axis indicates the content of each trait. Significance differences among the various genotypes are indicated by lowercase letter (*P* < 0.05; ANOVA)

In this study, KASP markers were developed from two SNPs. Specifically, *Whaas16493* was derived from the SNP which underlying the overlapping region of the QTL cluster *1DL-2* for glutenins and for gluten aggregation properties reported by our previous study) [19], whereas *Whaas115399* was located in the QTL cluster regions *6AS-3* (Table S7). These markers were used for evaluating the contribution of these regions to total glutenin and its fraction contents in the RIL population and in a set of 207 natural varieties (Tables S8-S10). In the RIL population, the lines with *Whass16493*-G and *Whass115339*-A had significantly higher total glutenin and its fraction contents than the lines with *Whass16493*-A and *Whass115339*-G which was as expected considering the additive effect of two parents (Table S9). Moreover, some lines harbored recombined genotypes in *Whass16493* and *Whass115339*. The lines with *Whaas16493*-A + *Whaas115399*-G, *Whaas16493*-A + *Whaas115399*-A, *Whaas16493*-G + *Whaas115399*-G or *Whaas16493*-G+ *Whaas115399*-A differed significantly in terms of the total glutenin and LMW-GS, Dx and Dy contents (Table S9). An evaluation of the genetic effects in the natural population revealed *Whass16493* influenced the content of glutenin fractions more than *Whaas115399*. Additionally, there was no significant difference in the effects of the *Whaas115399*-A and *Whaas115399*-G alleles among the varieties (Fig. 6, Tables S8 and S10). However, similar to the effects observed in the RIL population, the glutenin fraction content was higher for *Whass16493*-A than for *Whaas16493*-G (Fig. 6, Tables S8-10).

## Discussion

Although the glutenin content is significantly influenced by environment, this trait is mainly controlled by genetic factors. We identified genetic loci controlling total glutenin and its fraction contents via a QTL analysis, which revealed 41 QTL distributed on 16 chromosomes in an F_6_ RIL population over the 2-year investigation. Eight QTL associated with the glutenin content were revealed on chromosomes 1A, 1B, 1D, 3A, 4A and 5D. In addition to *Glu1A*, *Glu1B* and *Glu1D*, which were previously characterized as functional loci controlling the HMW-GS content in wheat, we also detected other loci located on chromosomes 5A, 2B, 7A and 5D known to contribute to the HMW-GS content [16, 17]. The main QTL related to the LMW-GS were located on chromosomes 1A, 1B, 1D, 2B, 3A, 5D, 7A and 7D [16, 17]. As predicted, QTL controlling the HMW-GS content were detected on the long arm of chromosome 1D, whereas the QTL for the LMW-GS content were present on the short arm of chromosomes 1A and 1D. The QTL on 1AS and 1DL control both HMW- and LMW-GS. The major QTL cluster *1DL-2* between markers *Whass16441* and *Whass16657* revealed in the present study partially overlapped the QTL cluster associated with gluten aggregation between markers *Whaas16407* and *Whaas16588* which we detected in an earlier study in which the *Glu-D1* was considered as the most likely candidate gene for the QTL cluster [19]. However, QTL were not detected on 1AL and 1BL for HMW-GS or on 1BS for LMW-GS. Additionally, QTL for HMW-GS were also detected on 3AL, 6AS and 7BS, whereas QTL for LMW-GS were detected on 3AS, 3AL, 3DL, 4DL, 5AL, 5BL, 6AS, 7BS and 7DS. Although relatively few studies have systematically assessed genetic loci affecting Ax (and Ay), Bx, By, Dx and Dy content, QTL associated with Bx and Dy have previously been mapped on chromosome 1B, and QTL associated with Dx have been located on chromosomes 1B and 5A [17]. The QTL cluster *6AS-3*, which included QTL controlling seven glutenin fractions (i.e., except for By), was not identified by previous studies. A comparison of the protein content-related QTL revealed in common and tetraploid wheat indicated QTL were distributed on 2A, 2B, 3A, 4BS, 4D, 5AL, 6AS, 6BS, 7AS, 7BS, 7BL and 7D [20]. To date, no QTL has been detected in a physical position similar to that of the QTL cluster *6AS-3*. More interestingly, the QTL cluster *6AS-3* is a pleiotropic locus that controls the content of total glutenin, GMW-GS, LMW-GS, Ax, Bx, Dx and Dy, and contributes to 3.1%-13.8% of the phenotypic variation for these traits. The detection of the QTL cluster *6AS-3* may provide a promising approach for improving all glutenin fraction contents at the same time by manipulating a single locus.

The QTL controlling the total glutenin and its fraction contents tended to cluster at the same physical positions in the genome. The seven QTL clusters detected in this study contained 32 of the 41 QTL detected for individual traits. The genes underlying these QTL clusters may function to increase the contents of all glutenin fractions. Previous studies revealed that in addition to allelic variations of glutenin synthesis genes, transcription factors may play an important role in regulating the expression of genes related to glutenin and its fractions. For example, a G-box element located in the promoter region of HMW-GS *Dx5* can initiate gene-specific expression in the endosperm [21]. These elements can also be specifically recognized and interact with different transcription factors that regulate the expression of HMW-GS and LMW-GS genes. More specifically, SPA transcription factors in the bZIP family can bind to the GCN4-like motif in the LMW-GS gene promoter to increase the expression level of this gene in wheat [22]. Additionally, the NAC family transcription factor TaNAC100 significantly increases the expression level of genes located downstream of the *Glu-1* promoter [23]. The By15, Dx2 and Dy12 contents as well as the total HMW-GS content significantly increase in transgenic lines over-expressing TaNAC100 [23]. Epigenetic modifications are also an important molecular mechanism for the regulation of the glutenin synthesis genes [24]. The histone acetyltransferase TaGCN5 interacts with the TaGAMyB transcription factor and together they bind to the promoter region of the HMW-GS genes, causing an increase in gene expression [25]. Our present study identified two major QTL clusters: 1DL-2 contributing to all eight investigated traits and 6AS-3 contributing to seven of the investigated traits (the exception was By). Other QTL clusters affected more than three traits. The genes in these pleiotropic QTL clusters may not be known genes for HMW-GS or LMW-GS genes, but they may encode other factors, perhaps including transcription factors or divergent chromatin status. We conclude that the synthesis and regulation of glutenin and its fractions involve a complex spatially and temporally specific network that will need to be further investigation.

Of the three HMW-GS loci located in the homologous regions of chromosome 1 (*Glu-1*), *Glu-D1* had the biggest impact on quality in wheat [26, 27]. The compositions of the subunits encoded by *Glu-D1* exert divergent effects on viscoelasticity and strength of dough [28, 29]. The rank order of the contribution of different subunits to dough strength is commonly assumed to be as follows: Dx5 + Dy10 > Dx2 + Dy12 > Dx3 + Dy12 > Dx4 + Dy12 [30]. An earlier study indicated that the bread-baking quality is greater for Dx2 + Dy12 than for Dx5 + Dy10 [31]. This phenomenon may derive from the higher content of Dx2 + Dy12 subunits according to the higher accumulation rate of the polymer and the higher proportion of polymer protein relative to monomer protein in the grain, which confers stronger gluten qualities [32]. Although the x- and y- type subunits of HMW-GS are tightly linked, there are also varieties containing recombinant subunits [31]. The recombinant subunit Dx5 + Dy12 confers higher baking quality than that of Dx5 + Dy10 and Dx2 + Dy12 [30, 32]. In the *Glu-B1* and *Glu-A1* loci, the contributions of different subunit compositions are as follows: Bx17 + By18 > Bx13 + By16 > Bx7 + By9 > Bx7 + By8 > Bx6 + By8 and Ax2* > Ax1 > Null, respectively [33–35]. In the present study, subunit-specific QTL for Ax (2BS-1, 3BL, 4AS, 4AL-1 and 4AL-2), Dx (1AS-2, 1BL-2 and 7AS-2), QDy (1DS-3, 5AL-2, 6AS-2), QBy (3DL-1, 7AS-1) and Bx (6AS-1) were detected. These results provide a possibility for selecting and creating germplasm harboring specific recombinant subunits as required. Production of lines with different x- and y- subunits through pyramiding of subunit-specific genetic loci may be facilitated by molecular markers for the specific glutenin fraction variants.

## Conclusions

In the present study, forty-one additive QTL and forty-three pairs of E-QTL associated with total glutenin and glutenin fraction contents were detected in a RIL population in four environments across two years. The detected QTL anchored to seven clusters which controlling more than three traits, whereas the QTL cluster *6AS-3* was recognized as a novel genetic locus. Two KASP markers for the two main QTL clusters *1DL-2* and *6AS-3* which can be used to effectively evaluate the content of glutenin fractions in the progeny of the two original parents as well as in natural wheat varieties. In practice, it is preferable to have more information about variants present for each gene related to quality in individual lines and to be able to evaluate the contributions of these variants to quality improvement. These two KASP markers can be used for marker-assisted selection of varieties with high glutenin fraction contents and for selecting individuals at the early developmental stages without needing to phenotype mature plants. This will increase the efficiency of selection and facilitate the creation of elite lines with high glutenin subunit contents for quality improvement in wheat.

## Materials and methods

### Plant materials and growing environments

The mapping population consists of 196 F_6_ Recombinant Inbred Lines (RILs) derived from two common wheat cultivars, Luozhen No.1 and Zhengyumai9987, which were originally provided by the Luohe Academy of Agricultural Sciences and the Youbang Crop Breeding Institute in Zhengzhou, respectively [19]. The two parents are divergent in quality related traits, not only in composition of glutenin but also in the quantity of each fraction of storage protein.

The RIL population was planted in the field experimental station of Henan Academy of Agricultural Science in Yuanyang (YY, E113°97′, N35°05′) and Yanjin (YJ, E114°36′, N35°10′) in 2017 – 2018, and in Yuanyang and Shangqiu (SQ, E115° 65′, N34° 45′) in 2018 – 2019. These cities are located in Henan province, which is the main wheat growing region in China. Each line was planted in a plot with two rows of 2 by 0.3 m, with 10 cm spaces between adjacent plants. Seeds were sown in October and the plants were harvested in May of the next year with normal treatments during the whole developmental period. A panel of 207 cultivars, collected from the Henan Province Crop Germplasm Bank and The International Maize and Wheat Improvement Center (CIMMYT) [36, 37] were also planted in the same environments in order to investigate whether the RIL results to a wider panel of cultivars. The authors declare the total permissions to use the collections.

### Glutenin extraction

The glutenins were extracted from 45 mg flour according to a published protocol [38] of with minor modifications. During the extraction procedure, 7% N-propanol (with 0.3M NaI), 70% ethanol and 50% isopropanol were used sequentially and the liquid glutenin extractions were filter-sterilized. Two replicates of each line were prepared for extraction.

### Measuring glutenin quantity and fractions

A reversed-phase high-performance liquid chromatography (RP-HPLC) system (Waters E2695+2998DAD,Waters Corporation, MA USA) with chromatographic column Vydac 218TP C18 (250mm × 4.6mm) was used for measuring glutenin quantity and fractions within 200 μl extractions [39]. The parameters were set as follows: the elution flow rate of elution was 0.8 ml/minute, the elution gradient was 0-10 minutes, 90% elution A (0.06% TFA solution in ddH_2_O) and 10% elution B (0.05% TFA solution in acetonitrile); elution A was linear decreasing to 35% within 10-65minutes, and the column temperature was 60°C. The content of each fraction was calculated according to the area value of corresponding peaks as follows:

$$\mathrm{Yu}=\frac{Tu\ast 100}{M\ast \left(1-X\right)}$$

Yu (10^6^AU/mg): content of each fraction; M (mg): weight of sample for extraction; X (%): water content of wheat flour measured by near infrared spectrum analyzer; Tu (AU): the peak area of each fraction; *100: 10 μl extraction was run on the RP-HPLC while the total extraction was 1 ml.

### Data analysis

Statistical analysis was conducted using various sets of tools or software packages. Significance was calculated by the *t*-test module of Microsoft Office Excel. The phenotypic description parameters, such as variation, mean value, standard deviation, coefficient of variation, were analyzed using the “psych” package of R (version 3.5.3) (R Core Team 2020). BLUP (Best Linear Unbiased Prediction) and broad-sense heritability for each trait were calculated by the “Lme4” package in R. Phenotypic variations and correlations were analyzed by SAS 9.2 and IBM SPSS Statistics 22, respectively.

### Construction of the genetic map

The SLAF (Specific Locus Amplified Fragment Sequencing) technology was used for mining SNPs (Single Nucleotide Polymorphisms) between the two parents and in the RILs. The mLOD between SLAF tags was calculated and used to distinguish different linkage groups. The HighMap software was used for evaluating the genetic distance between SLAF tags according to Maximum Likelihood Estimation and arranging their order in each linkage group. A total of 1,544.06 M reads were obtained, of which 90.37% of reads were pair-end aligned with the reference genome sequence [40]. Finally, a genetic map was constructed with 8,942 total SNPs located on 21 linkage groups. The total genetic length was 3,140.54 cM and the average genetic distance between two adjacent markers was 0.35 cM.

### Analysis of QTL and epistatic QTLs

The software package “QTL.gCIMapping.GUI” in R was used for QTL mapping. The genetic map constructed by SLAF tags and all RIL phenotype data was imported into the software, which was run as follows: data format = GCIM, logarithm of the odds (LOD) score = 2.5 for the random model used for QTL screening. The additive-by-additive E-QTL were analyzed using QTL IciMapping software. More specifically, the ICIM-EPI method was used with the mapping parameters set as follows: step (cM): 25; probability in stepwise regression: 0.0001; LOD Threshold 1.000 [41, 42].

### Candidate gene analysis for the two main QTL clusters

The confidence intervals of the QTL clusters which can be identified with seven or more surveyed traits were selected to detect the candidate genes. Based on the physical position of flanking markers according to the Chinese Spring reference genome (IWGSC v1.1), all genes harbored in the confidence intervals were regarded as the candidate genes. All the annotated candidate genes were categorized through Gene Ontology (GO), the Kyoto Encyclopedia of Genes and Genomes (KEGG), eukaryotic orthologous groups (KOG) and the Swiss-prot database analysis.

### KASP marker development

Genomic sequences with a length of 100 bp on the 5′ and 3′ strands surrounding the target SNP were extracted. Two allele-specific primers were designed carrying the FAM: (5′-TGAAGGTGACCAAGTTCATGCT3-′) and HEX: (5′- GAAGGTCGGAGTCAACGGATT 3-′) sequences at the 5′ end. The target SNP was anchored at the 3′ end of each primer. The sequences from which the target SNPs were derived were used to identify homologous sequences via BLAST using the website EnsemblPlants database (http://plants.ensembl.org/index.html). Five to eight sequences with the highest homology were selected. These sequences were aligned and the conserved regions were used for designing allele-specific primer pairs. The PCR reactions were prepared using the KASP Assay mixture and a Bio-Rad CFX Maestro was used for fluorescence detection and data analysis.

## Supplementary Information

**Additional file 1: Table S1** Phenotypic data of all the traits under all the four environments. **Table S2** Phenotypic variation of two parents and RIL population in four environments and the average value. **Table S3** The correlation coefficient between different traits. **Table S4** QTL associated with content of glutenin and its fractions in a bread wheat RIL population across four environments. **Table S5** E-QTL for total glutenin and its fractions in four environments of the RIL population. **Table S6** List of 164 annotated genes located in the two main QTL clusters of *1DL-2* and *6AS-3*. **Table S7** Sequence for KASP markers development. **Table S8** Genotyping by two KASP markers and glutenin and its fractions content in natural accessions. **Table S9** Additive effect analysis of two KASP markers for glutenin content and fractions in the RIL population. **Table S10** Additive effect analysis of the significant SNPs on major QTL clusters associated with content of glutenin subunits fractions in natural population.

**Additional file 2: Figure S1.** QTL detected for the content of total glutenin. E1, E2, E3 and E4 represent the environments of Yuanyang (2018), Yanjin (2018), Yuanyang (2019), Shangqiu (2019), respectively. BLUP represents the QTL analysis with best linear unbiased prediction. The red peak represent the QTL screened. The name of each QTL assigned according to nomenclature were labeled. **Figure S2**. QTL detected for HMW-GS content. Legends accordingly with Figure S1. **Figure S3**. QTL detected for LMW-GS content. Legends accordingly with Figure S1. **Figure S4**. QTL detected for Ax content. Legends accordingly with Figure S1. **Figure S5**. QTL detected for Bx content. Legends accordingly with Figure S1. **Figure S6**. QTL detected for By content. Legends accordingly with Figure S1. **Figure S7**. QTL detected for Dy content. Legends accordingly with Figure S1. **Figure S8**. QTL cluster for glutenin and its fractions detected in 1AS-1 region. Curves with different colors indicated different traits. Molecular markers around the peak of the cluster and their corresponding genetic position were labeled. The major locus for controlling glutenin content, Glu-D1 which coloured with purple, was mapped in this cluster. Two SNPs flanking the cluster which were used for KASP marker development coloured in red. **Figure S9**. QTL cluster for glutenin and its fractions detected in 1BL-1 region. Legends accordingly with Figure S8. **Figure S10**. QTL cluster for glutenin and its fractions detected in 1DL-3 region. Legends accordingly with Figure S8. **Figure S11**. QTL cluster for glutenin and its fractions detected in 3AS-2 region. Legends accordingly with Figure S8. **Figure S12**. GO analysis of the annotated candidate genes in the two main QTL clusters. **Figure S13**. KEGG analysis of the annotated candidate genes in the two main QTL clusters. **Figure S14**. KOG analysis of the annotated candidate genes in the two main QTL clusters.

## Data Availability

All data generated or analyzed during this study are included in this published article [and its supplementary information files] and the raw SLAF sequencing data can be found in Genome Sequence Archive (https://bigd.big.ac.cn/gsa/browse/CRA003543).

## Abbreviations

RILs: Recombinant Inbred Lines
QTL: Quantitative Trait Loci
KASP: Kompetitive Allele-Specific PCR
HMW-GS: High Molecular Weight Subunits
LMW-GS: Low Molecular Weight Subunits
LOD: Logarithm of the Odds
BLUP: Best Linear Unbiased Prediction
