# Supplementary material for: Quantitative traits loci mapping and molecular marker development for total glutenin and glutenin fraction contents in wheat
Source: BMC Plant Biol. 2021 Oct 6;21:455. doi: 10.1186/s12870-021-03221-0 (PMC8493754; doi:10.1186/s12870-021-03221-0)

**Figure S1 QTL detected for the content of total glutenin**

E1, E2, E3 and E4 represent the environments of Yuanyang (2018), Yanjin (2018), Yuanyang (2019), Shangqiu (2019), respectively. BLUP represents the QTL analysis with best linear unbiased prediction. The red peak represent the QTL screened. The name of each QTL assigned according to nomenclature were labeled.

**Figure S2 QTL detected for HMW-GS content**

Legends accordingly with Figure S1.

**Figure S3 QTL detected for LMW-GS content**

Legends accordingly with Figure S1.

**Figure S4 QTL detected for Ax content**

Legends accordingly with Figure S1.

**Figure S5 QTL detected for Bx content**

Legends accordingly with Figure S1.

**Figure S6 QTL detected for By content**

Legends accordingly with Figure S1.

**Figure S7 QTL detected for Dy content**

Legends accordingly with Figure S1.

**Figure S8 QTL cluster for glutenin and its fractions detected in 1AS-1 region**

Curves with different colors indicated different traits. Molecular markers around the peak of the cluster and their corresponding genetic position were labeled. The major locus for controlling glutenin content, Glu-D1 which coloured with purple, was mapped in this cluster. Two SNPs flanking the cluster which were used for KASP marker development coloured in red.

**Figure S9 QTL cluster for glutenin and its fractions detected in 1BL-1 region**

Legends accordingly with Figure S8.

**Figure S10 QTL cluster for glutenin and its fractions detected in 1DL-3 region**

Legends accordingly with Figure S8.

**Figure S11 QTL cluster for glutenin and its fractions detected in 3AS-2 region**

Legends accordingly with Figure S8.

**Figure S12 GO analysis of the annotated candidate genes in the two main QTL clusters**

**Figure S13 KEGG analysis of the annotated candidate genes in the two main QTL clusters**

**Figure S14 KOG analysis of the annotated candidate genes in the two main QTL clusters**

**Figure S1**


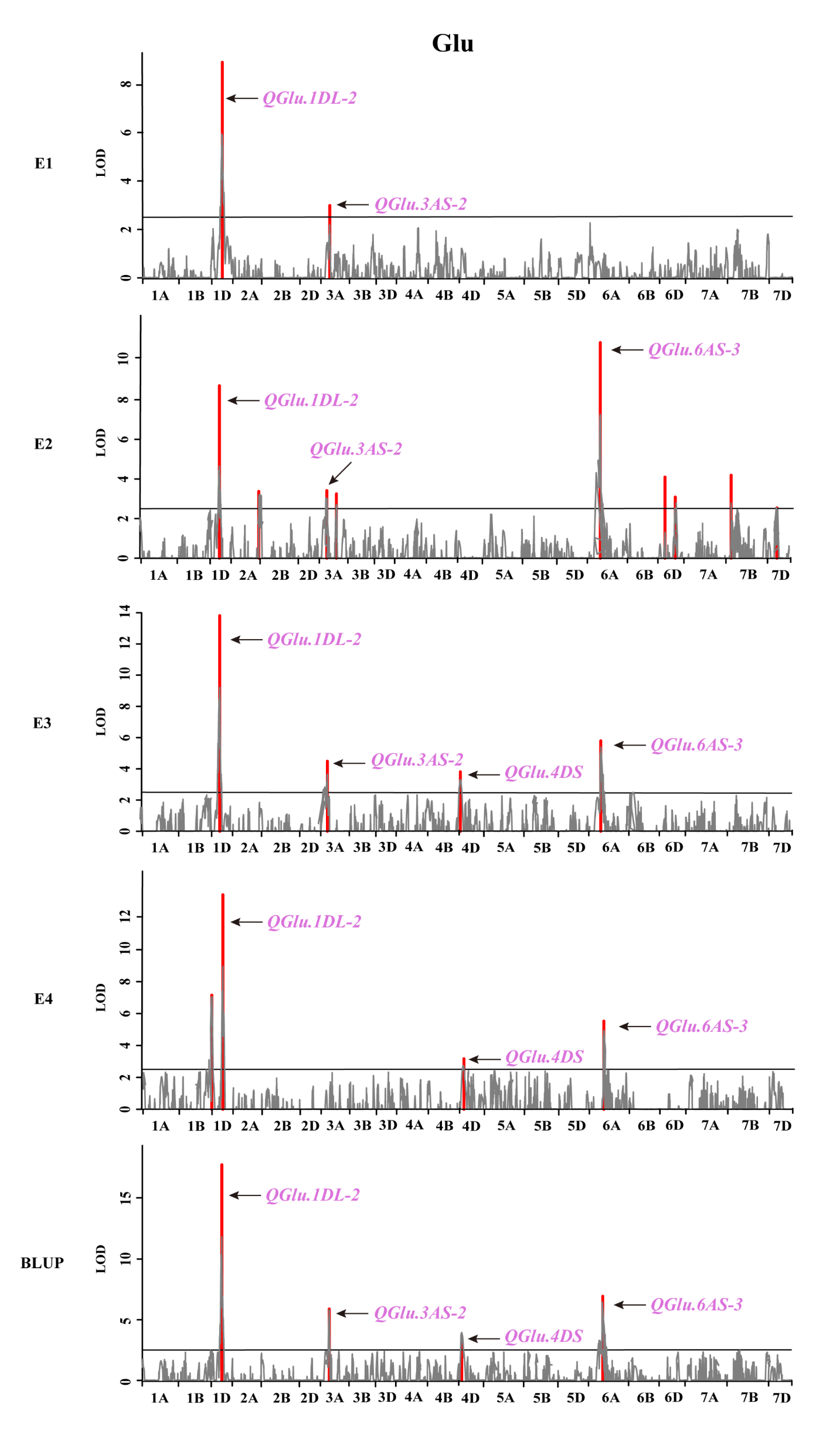


**Figure S2**


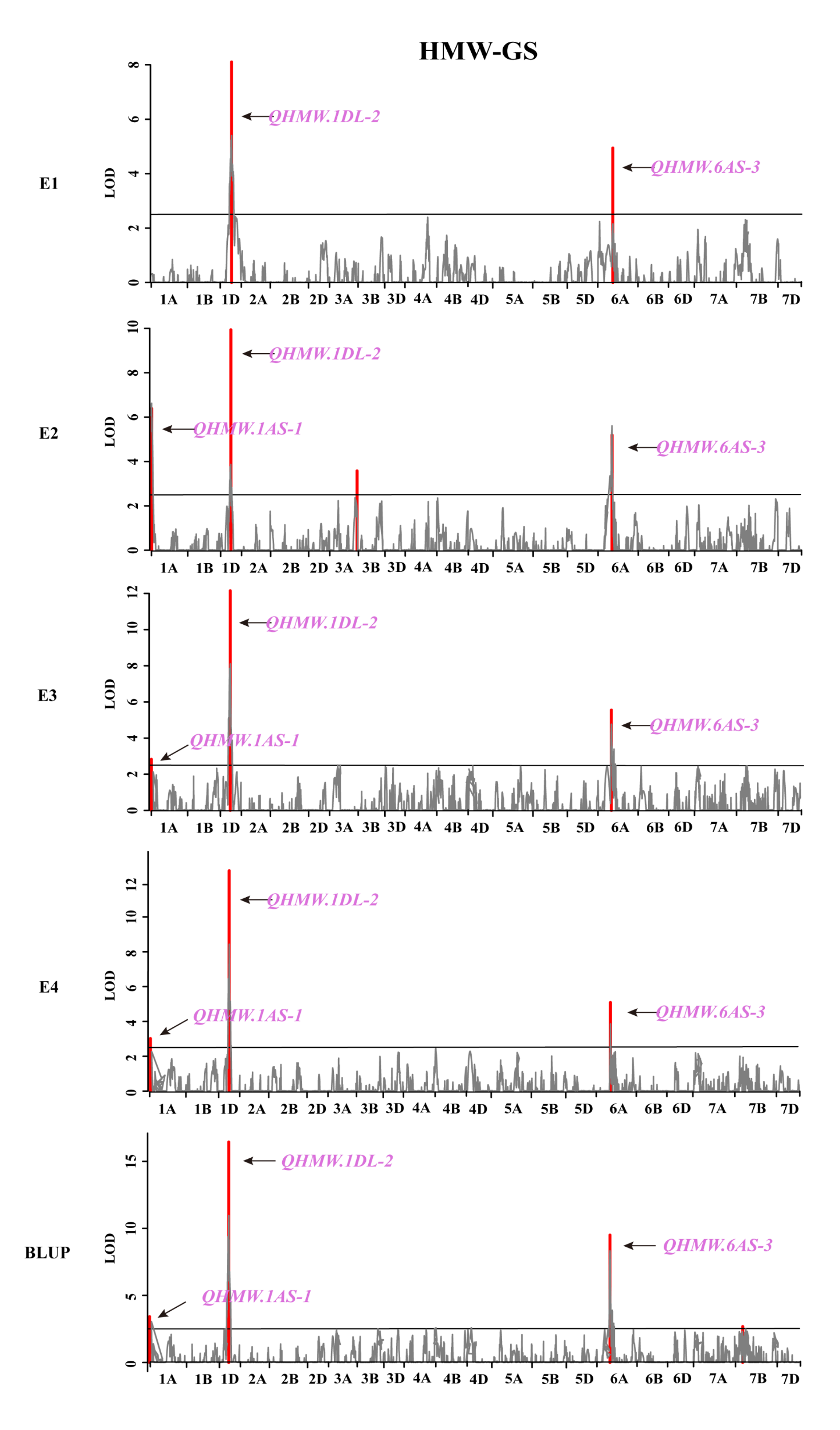


**Figure S3**


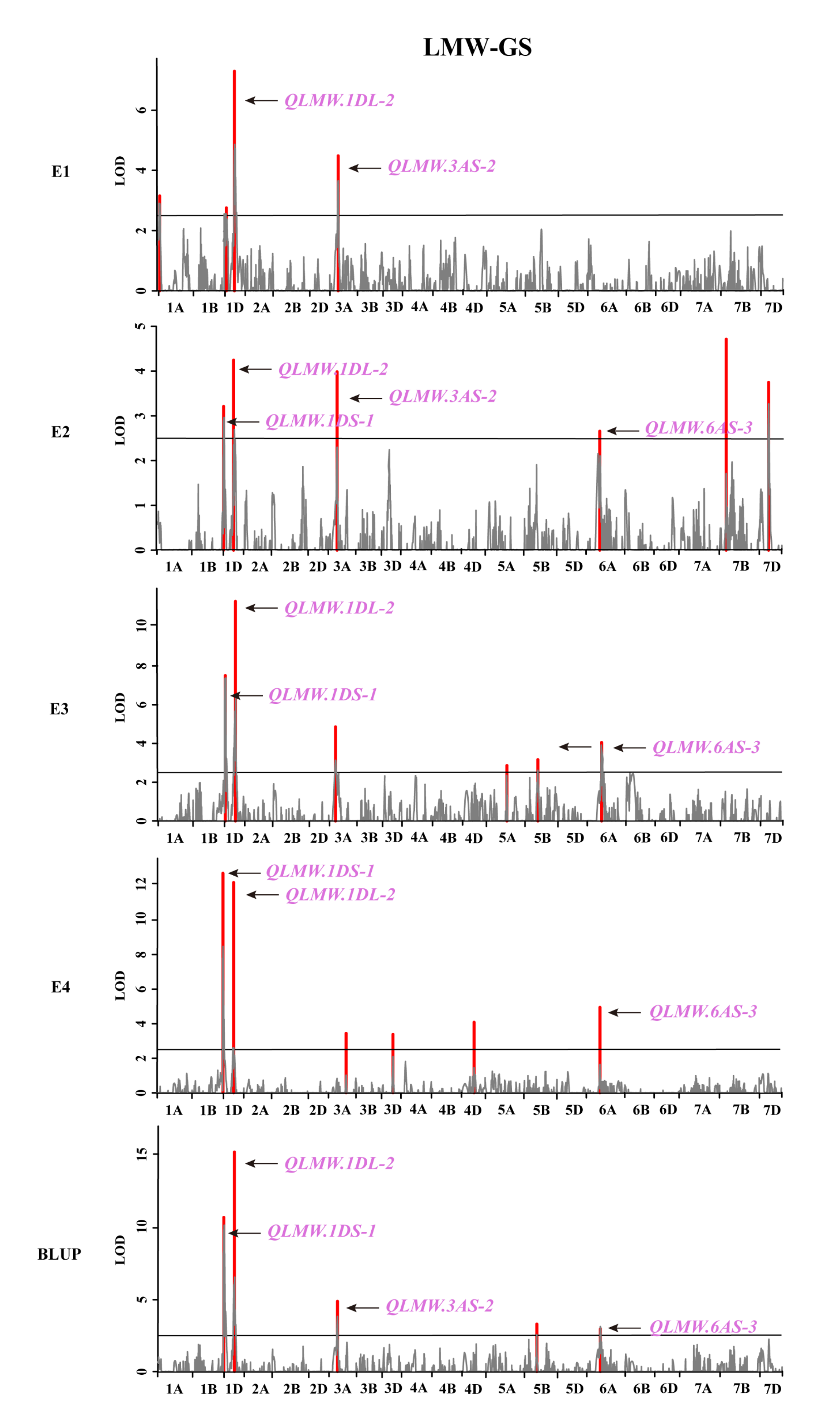


**Figure S4**


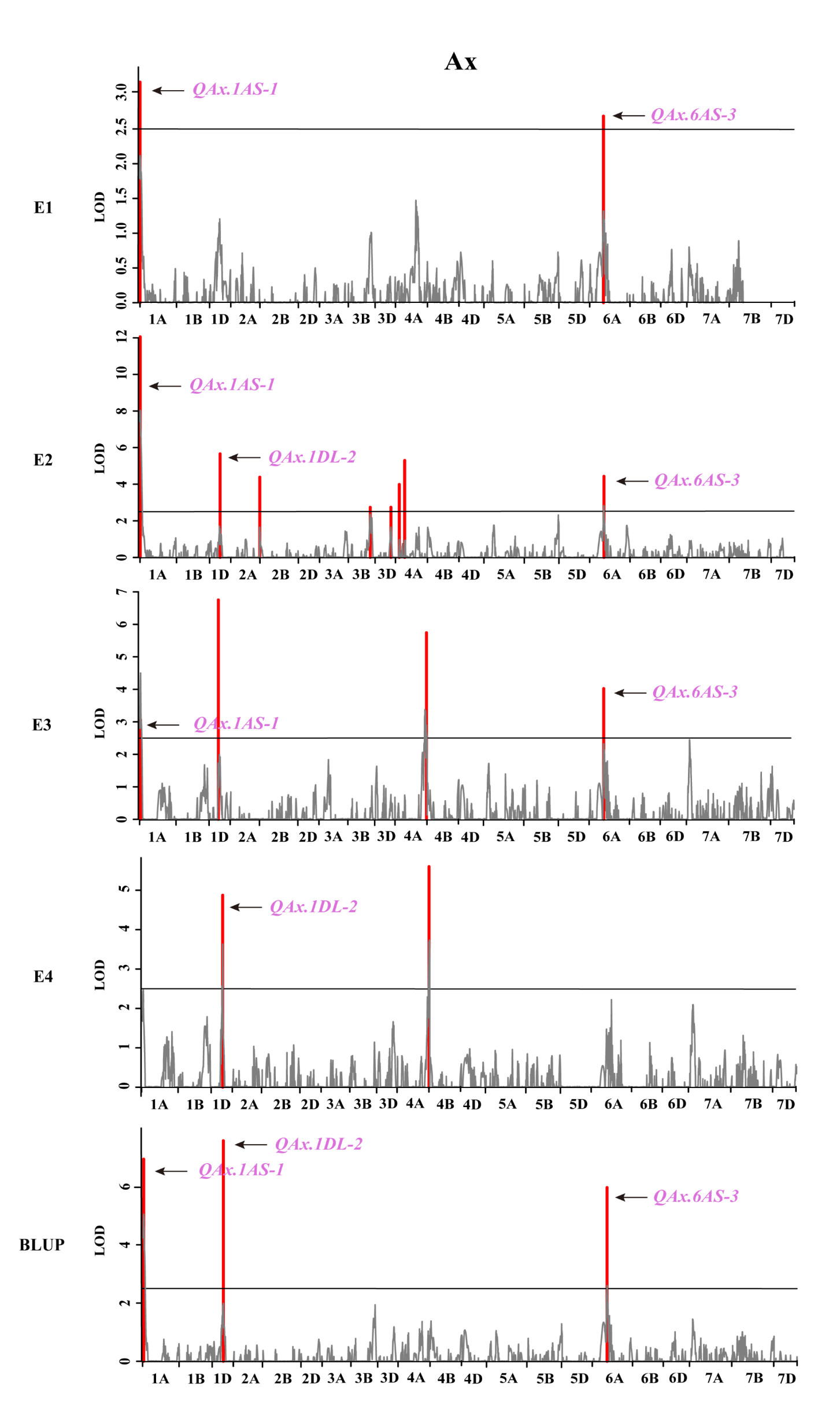


**Figure S5**


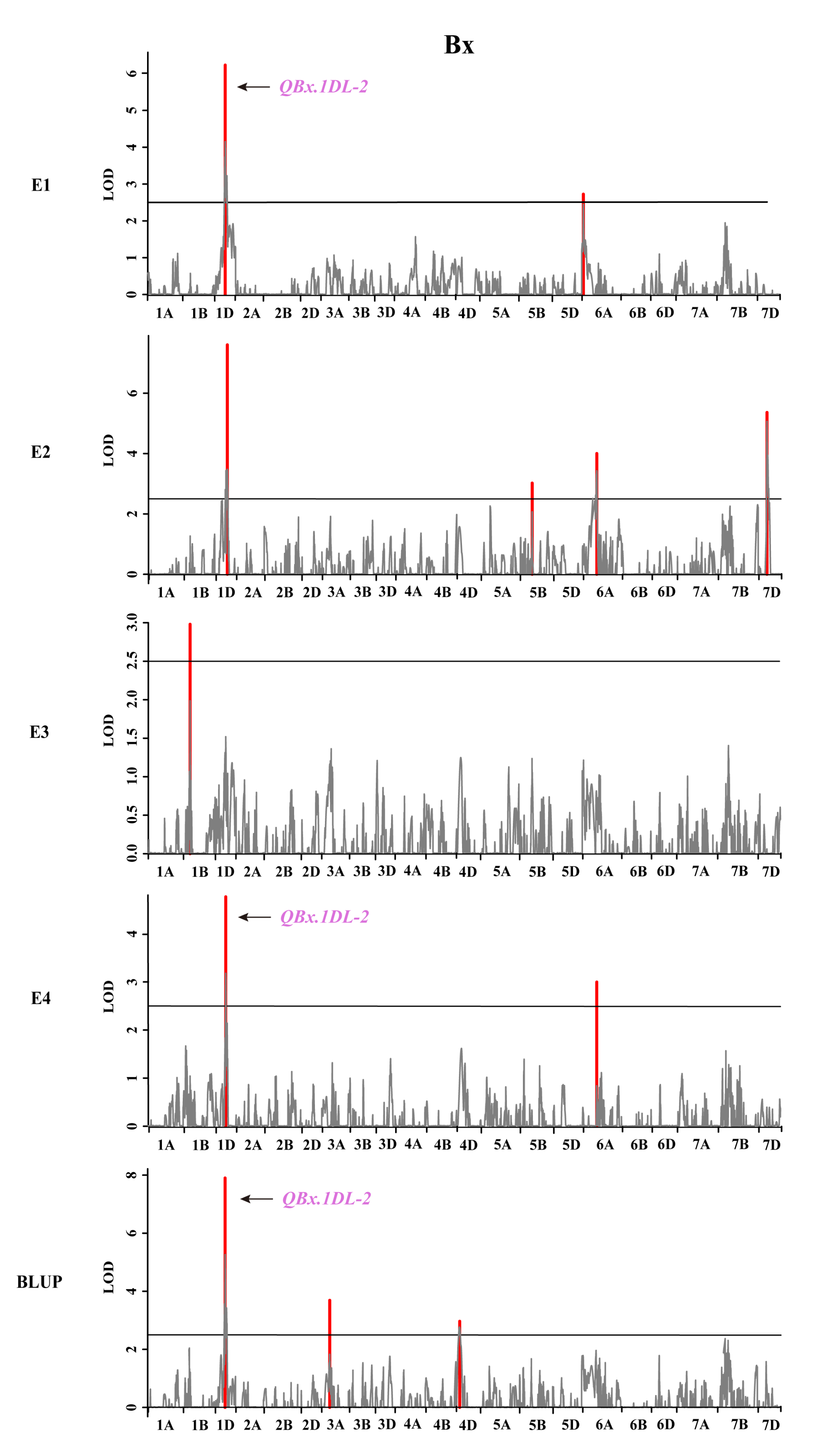


**Figure S6**


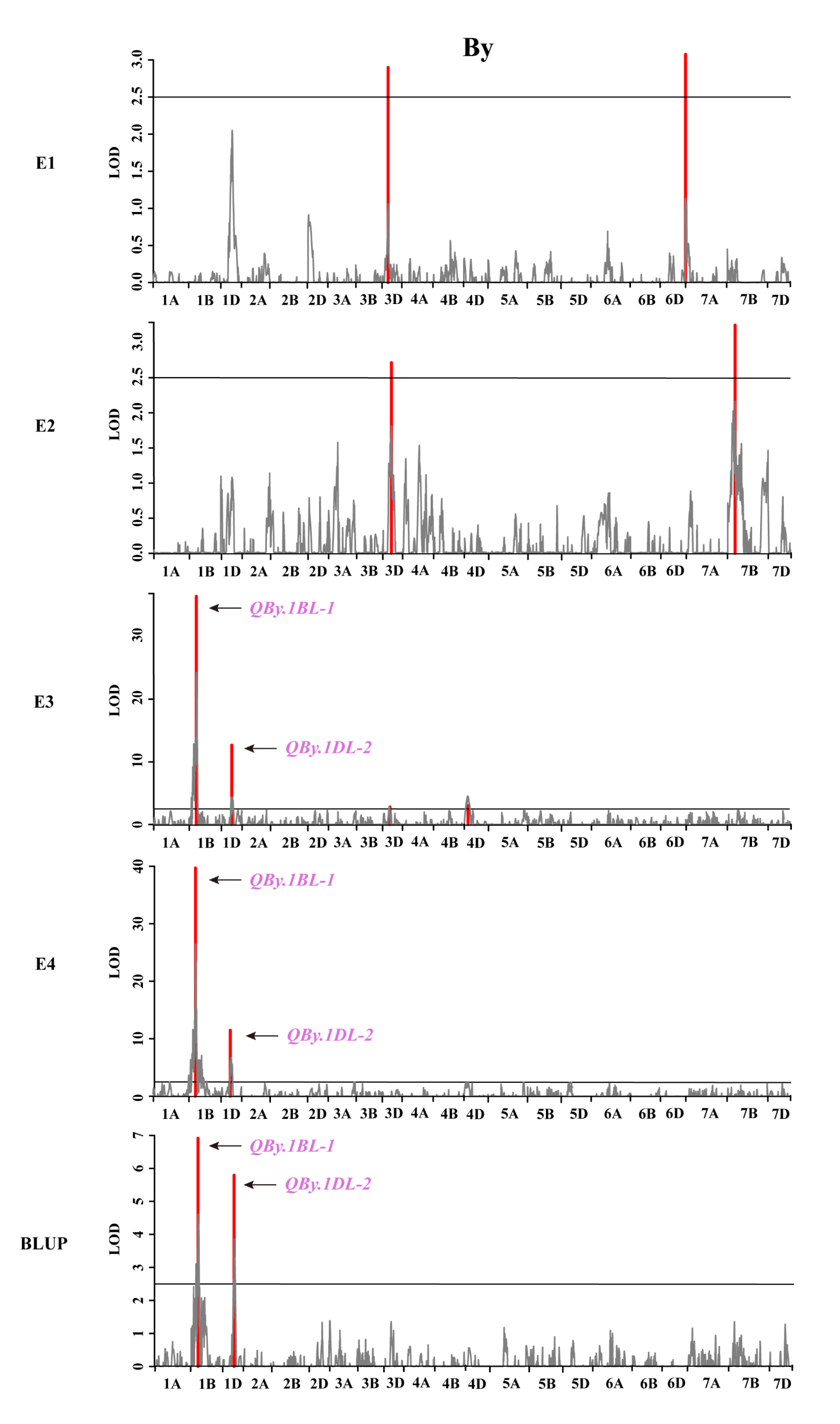


**Figure S7**


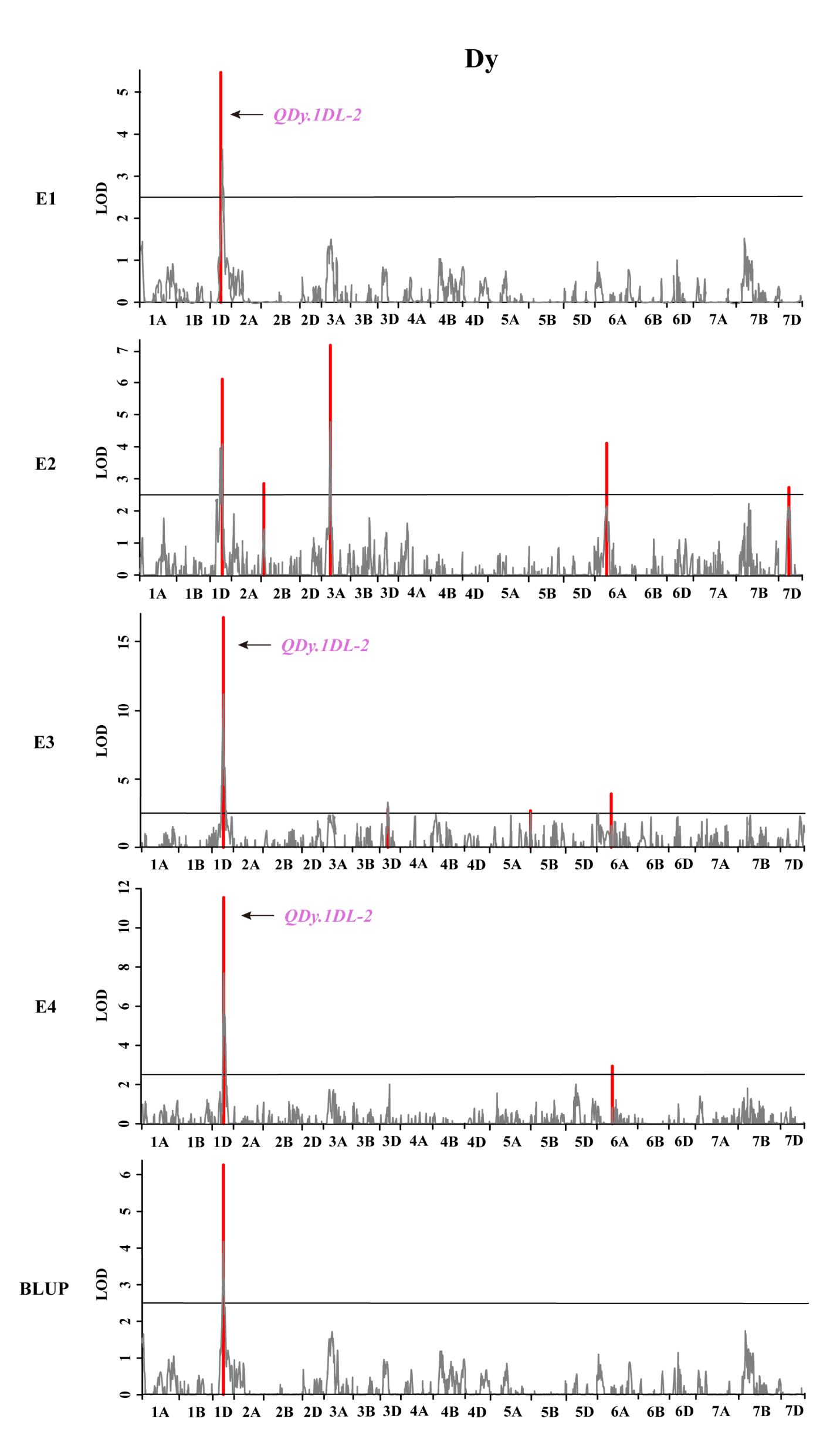


**Figure S8**

**1AS-1**

**
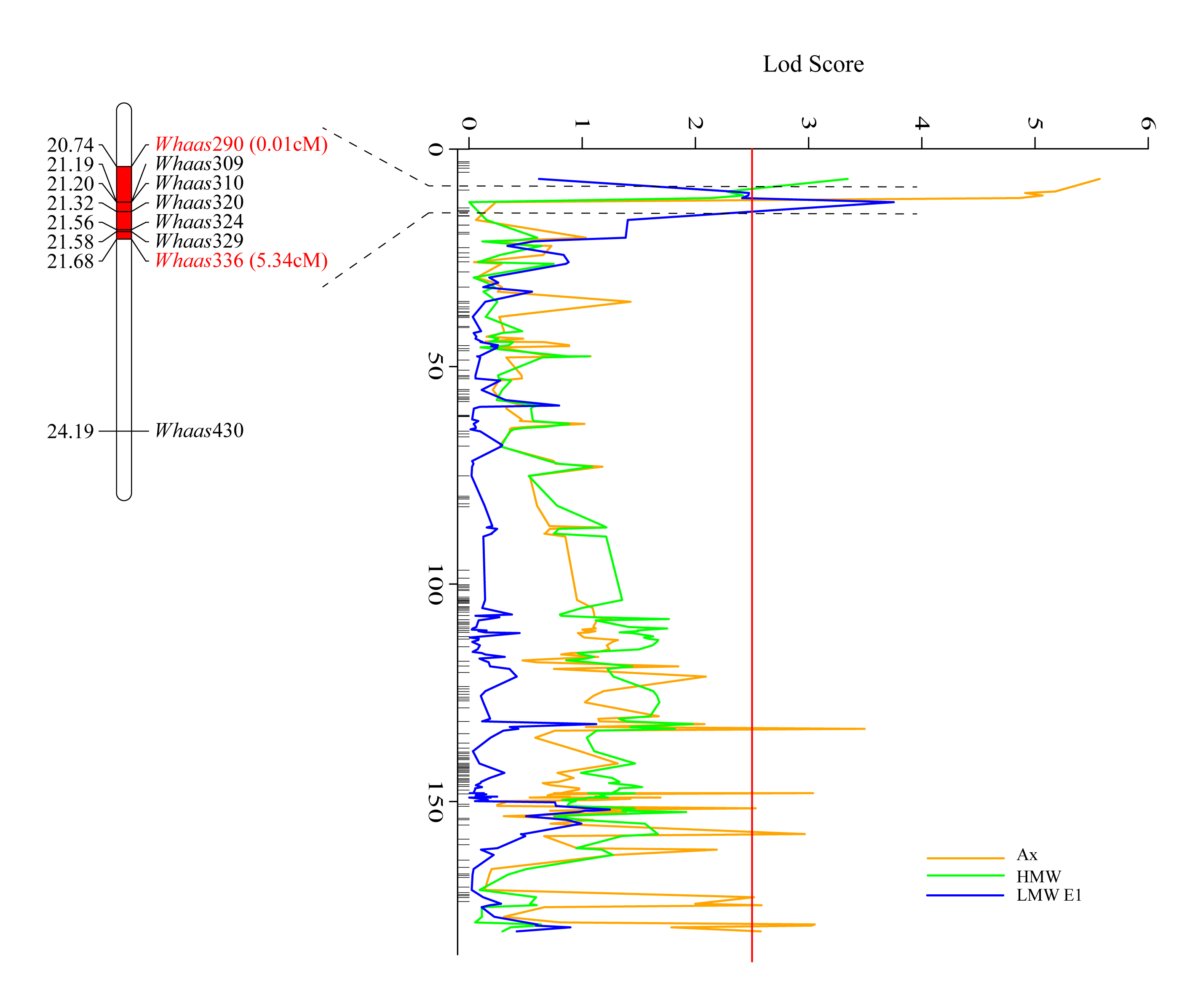
**

**Figure S9**

**1BL-1**

**
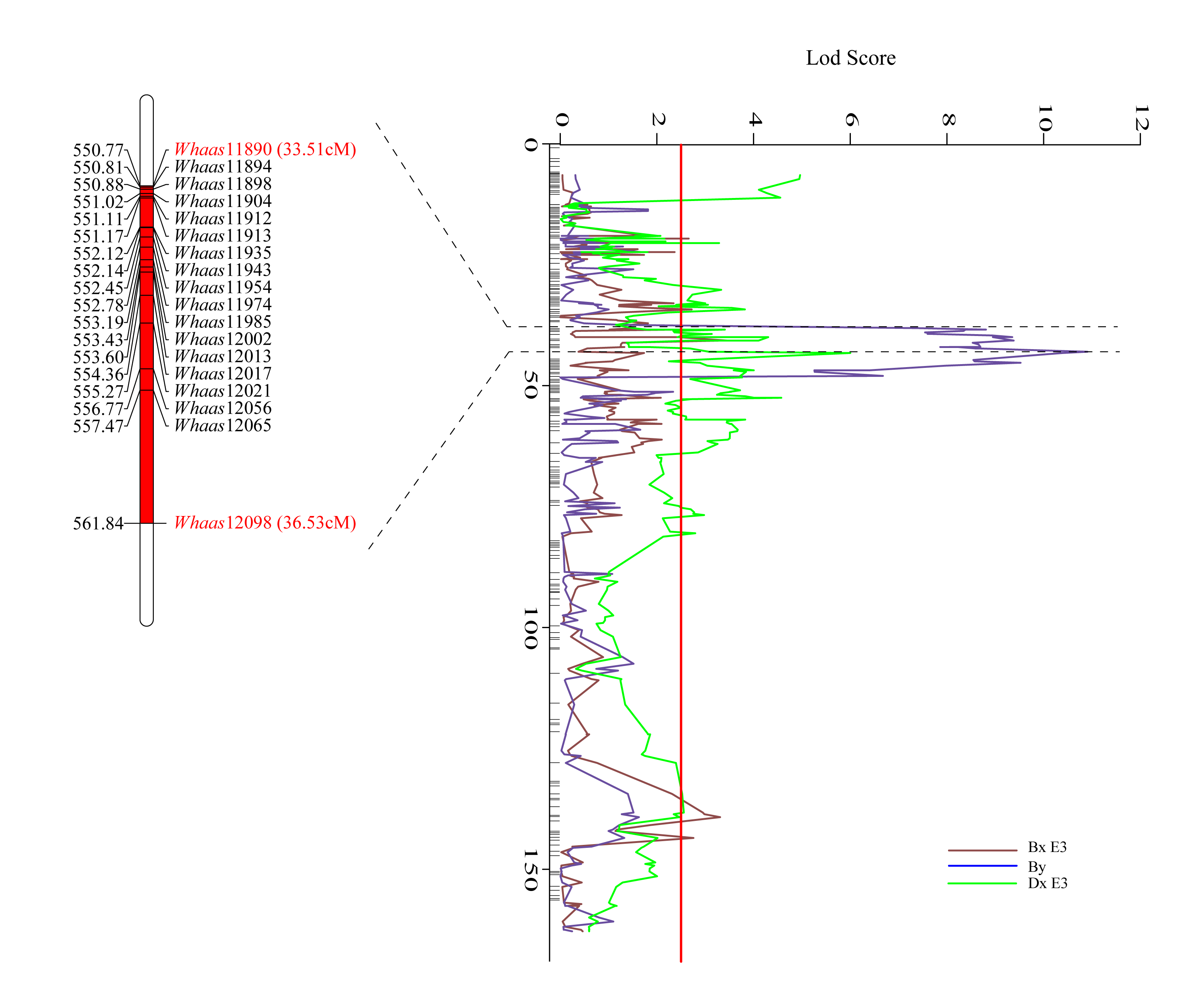
**

**Figure S10**

**1DL-3**

**
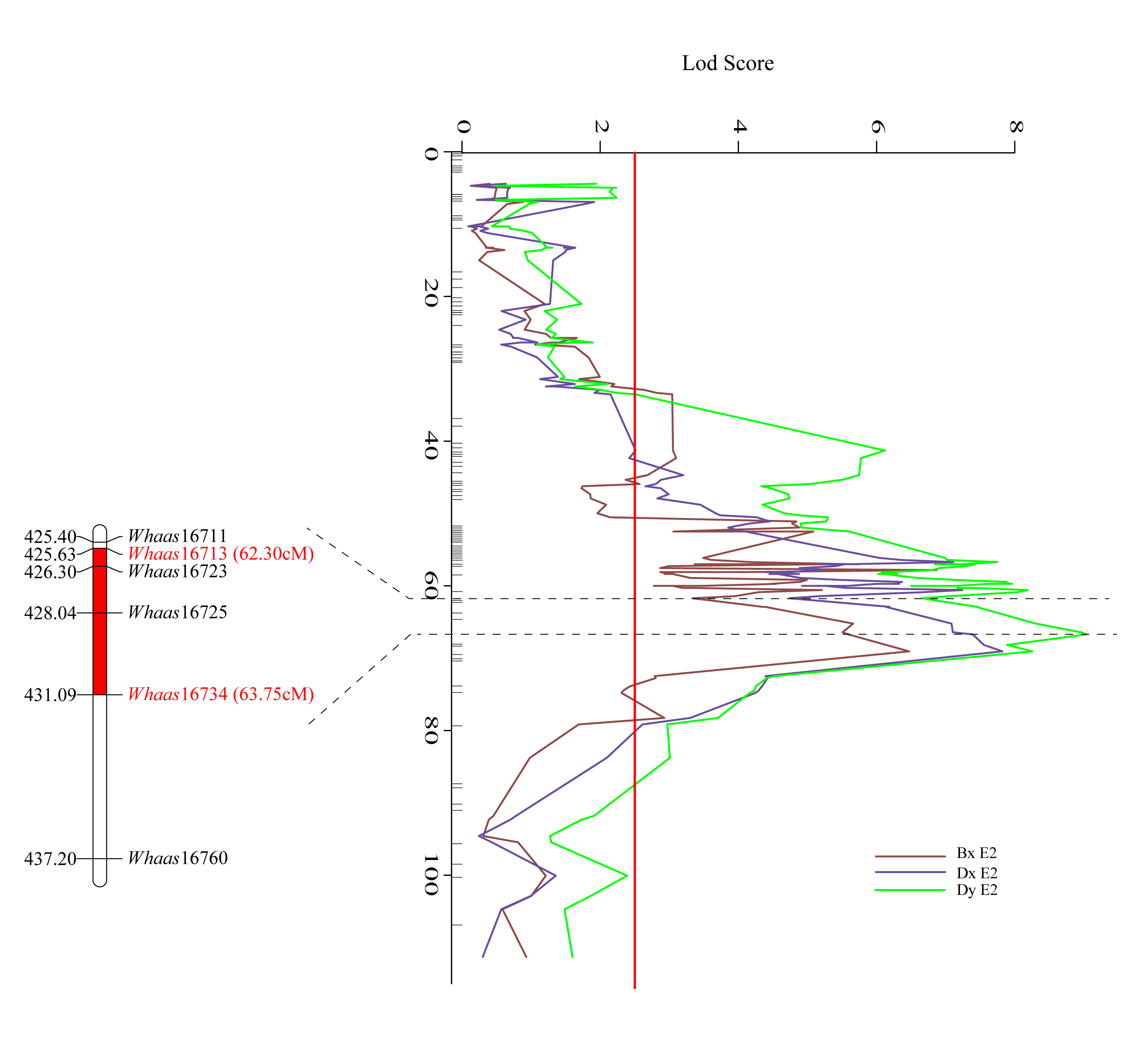
**

**Figure S11**

**3AS-2**

**
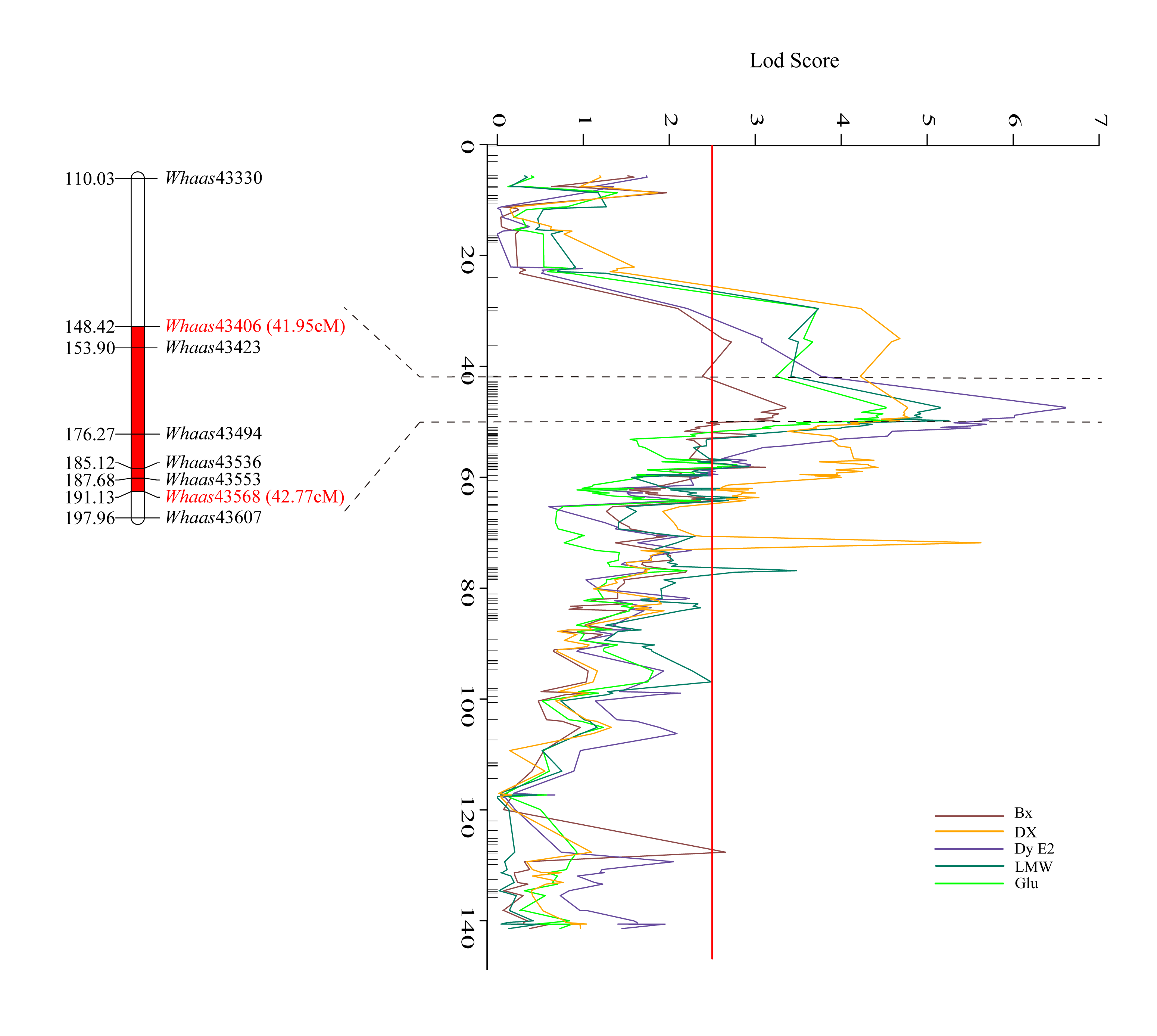
**

**Figure S12**


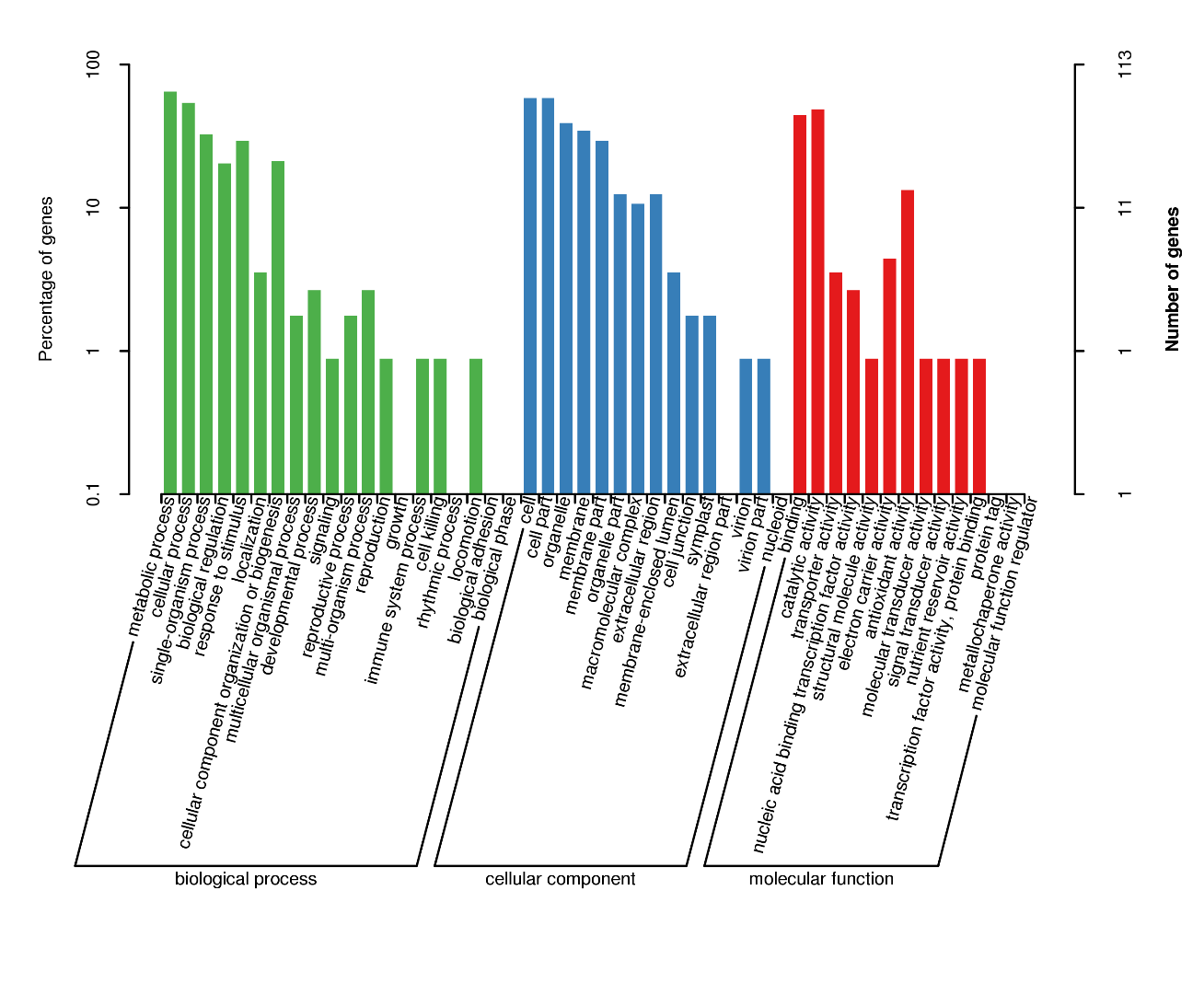


**Figure S13**


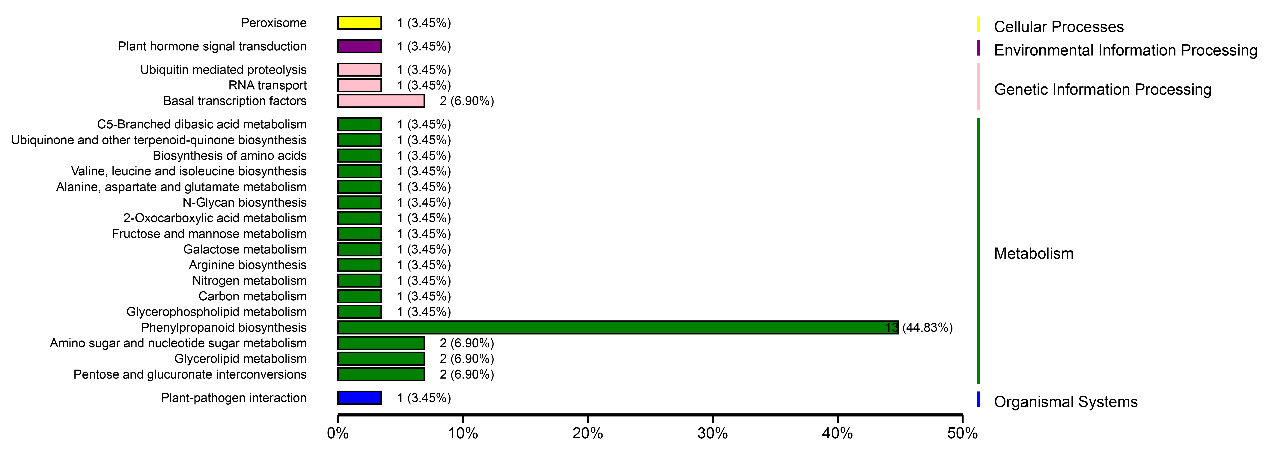


**Figure S14**


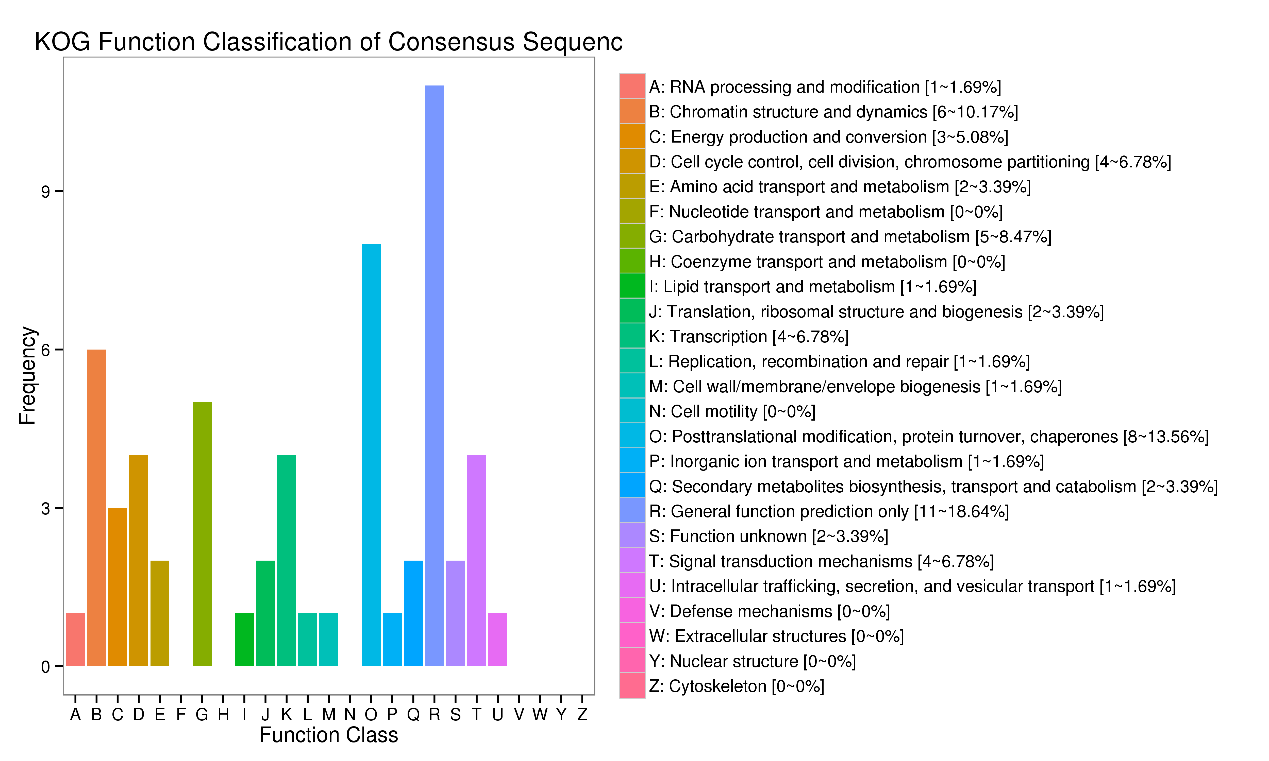

Supplement: Supplementary file 2 — Additional file 2: Figure S1. QTL detected for the content of total glutenin. E1, E2, E3 and E4 represent the environments of Yuanyang (2018), Yanjin (2018), Yuanyang (2019), Shangqiu (2019), respectively. BLUP represents the QTL analysis with best linear unbiased prediction. The red peak represent the QTL screened. The name of each QTL assigned according to nomenclature were labeled. Figure S2. QTL detected for HMW-GS content. Legends accordingly with Figure S1. Figure S3. QTL detected for LMW-GS content. Legends accordingly with Figure S1. Figure S4. QTL detected for Ax content. Legends accordingly with Figure S1. Figure S5. QTL detected for Bx content. Legends accordingly with Figure S1. Figure S6. QTL detected for By content. Legends accordingly with Figure S1. Figure S7. QTL detected for Dy content. Legends accordingly with Figure S1. Figure S8. QTL cluster for glutenin and its fractions detected in 1AS-1 region. Curves with different colors indicated different traits. Molecular markers around the peak of the cluster and their corresponding genetic position were labeled. The major locus for controlling glutenin content, Glu-D1 which coloured with purple, was mapped in this cluster. Two SNPs flanking the cluster which were used for KASP marker development coloured in red. Figure S9. QTL cluster for glutenin and its fractions detected in 1BL-1 region. Legends accordingly with Figure S8. Figure S10. QTL cluster for glutenin and its fractions detected in 1DL-3 region. Legends accordingly with Figure S8. Figure S11. QTL cluster for glutenin and its fractions detected in 3AS-2 region. Legends accordingly with Figure S8. Figure S12. GO analysis of the annotated candidate genes in the two main QTL clusters. Figure S13. KEGG analysis of the annotated candidate genes in the two main QTL clusters. Figure S14. KOG analysis of the annotated candidate genes in the two main QTL clusters. [file 12870_2021_3221_MOESM2_ESM.docx]
